# Comparative Outcomes of Open Radical Cystectomy vs. Robot-Assisted Approaches with Intracorporeal and Extracorporeal Urinary Diversion: A Meta-Analysis and Network Meta-Analysis of Perioperative and Quality of Life Outcomes

**DOI:** 10.3390/jcm13082421

**Published:** 2024-04-21

**Authors:** Rocco Simone Flammia, Leslie Claire Licari, Eugenio Bologna, Riccardo Mastroianni, Flavia Proietti, Gabriele Tuderti, Umberto Anceschi, Aldo Brassetti, Antonio Franco, Cosimo De Nunzio, Riccardo Autorino, Costantino Leonardo, Giuseppe Simone

**Affiliations:** 1 Department of Maternal-Child and Urological Sciences, Sapienza University Rome, Policlinico Umberto I Hospital, 00185 Rome, Italy; roccosimone92@gmail.com (R.S.F.); leslieclaire.licari@uniroma1.it (L.C.L.); 2 Department of Urology, “Regina Elena” National Cancer Institute, 00144 Rome, Italy; riccardo.mastroianni@ifo.it (R.M.); gabriele.tuderti@ifo.it (G.T.); umberto.anceschi@ifo.it (U.A.); aldo.brassetti@ifo.it (A.B.); costantino.leonardo@gmail.com (C.L.); puldet@gmail.com (G.S.); 3 Department of Urology, Rush University, Chicago, IL 60612, USA; antonio.franco@uniroma1.it (A.F.); ricautor@gmail.com (R.A.); 4 Department of Urology, Sant’Andrea Hospital, Sapienza University, 00185 Rome, Italy; cosimo.denunzio@uniroma1.it

**Keywords:** bladder cancer, ICUD, ECUD, minimally invasive, network meta-analysis

## Abstract

**Background:** To conduct a comprehensive systematic review and network meta-analysis of RCTs that compare outcomes of robot-assisted radical cystectomy (RARC) with intra- or extracorporeal urinary diversion (ICUD or ECUD) and the standard open approach (oRC). **Methods:** A systematic review identified RCTs including patients aged >18 years with non-metastatic bladder cancer treated with RARC (ICUD or ECUD) vs. oRC and reporting peri- and post-operative outcomes and quality of life (QoL) assessment. Standard and network metanalyses were performed. **Results:** Data from 1024 patients included in eight RCTs were analyzed. The standard meta-analysis found that RARC had longer OT, lower EBL, and a lower transfusion rate compared to oRC (all *p* < 0.001). No significant differences in terms of LOS between the ICUD vs. ECUD vs. ORC were recorded. RARC patients demonstrated better scores in fatigue, insomnia, pain, physical functioning, and role functioning—according to QoL assessment—compared to ORC at early follow-up, despite no difference at baselines. Finally, at network metanalysis, ICUD (OR = 0.74, *p* < 0.001) but not ECUD (OR = 0.92, *p* < 0.08) yielded a lower rate of high-grade 90-day complications compared to ORC despite longer OT (MD = 89.56, *p* = 0.0351). **Conclusions:** RARC represents a safe and feasible option to reduce perioperative bleeding with no definitive impact on LOS compared to ORC. Interestingly, ICUD may reduce the burden of 90-day complications to a greater extent than ECUD. Nonetheless, surgeons should be aware of the extended OT and steep learning curve of ICUD. Finally, RARC may provide some short-term benefits in terms of QoL, but more research is needed to determine its long-term effects.

## 1. Introduction

Radical Cystectomy (RC) with extended pelvic lymph node dissection (ePLND) represents the standard of care for muscle-invasive bladder cancer (MIBC) and high-risk non-muscle-invasive bladder cancer (NMIBC) refractory to intravesical treatment [1,2]. Although it represents a pivotal treatment in the management of these disease stages, RC is associated with considerable morbidity, with rates of high-grade complications reported between 13% and 20% [3,4]. In recent years, in an effort to reduce peri- and post-operative complications—mirroring trends observed in other urological oncological surgeries [5]—the adoption of robot-assisted radical cystectomy (RARC) has been gaining traction [6]. Multiple authors have reported their experiences in terms of case series, retrospective cohort studies, and, more notably, randomized controlled trials (RCTs) [7,8]. Studies such as the RAZOR and iROC trials, which are the most extensive multicentric RCTs to date, are currently considered landmark studies in this field [9,10].

A recent Cochrane meta-analysis reported a comparison of outcomes between Open Radical Cystectomy (ORC) and RARC. The two approaches were found to be comparable in terms of oncological outcomes, rates of major complications, quality of life (QoL), and positive margin rates; RARC is associated—albeit with levels of evidence varying from very low to moderate—with a reduction in minor complications, a decreased risk of blood transfusions, and a shorter hospital stay [11]. During RARC, the urinary diversion can be fashioned using either a totally intracorporeal (ICUD) or a hybrid open-minimally invasive extracorporeal (ECUD) approach. The adoption of the ICUD approach in RARC has been increasing annually by 11% since 2005 [12]. Various studies have compared RARC with ECUD and ICUD or examined each modality independently [13,14,15]. Some authors support the hypothesis that the wide abdominal incision, bowel manipulation, and exposure to room temperature—characteristics inherent to the ECUD approach—may diminish the benefits derived from a totally robotic, minimally invasive procedure. These theoretical advantages were supported by a recent meta-analysis concluding that patients receiving ICUD experienced comparable complications, superior perioperative outcomes, and similar oncological outcomes compared with ECUD [16]. However, this analysis incorporated both retrospective and prospective data, drawing its conclusions from subgroup analyses.

To date, there are no randomized trials comparing ECUD and ICUD, resulting in a lack of high-quality comparative studies between these two approaches during RARC. To bridge this gap, we have designed a systematic review and network meta-analysis comparing ORC and RARC with ECUD or ICUD. This study seeks to assess whether ICUD is superior to ECUD in perioperative outcomes and post-operative QoL, utilizing the comprehensive insights offered by network meta-analysis.

## 2. Materials and Methods

### 2.1. Search Strategy

We conducted a systematic review in line with the Preferred Reporting Items for Systematic Reviews and Meta-Analyses (PRISMA) guidelines [17]. The study protocol was registered in the International Prospective Register of Systematic Reviews (PROSPERO) database (Registration Number: CRD42023413190). We performed a systematic search of the literature via Medline/Pubmed, Web of Science, Scopus, Cochrane Library and ClinicalTrials.gov databases up to September 2023. The full search strategy is provided in the Supplementary Material (Table S1).

The following PICOS criteria were used:

P (patients): Patients aged >18 years with non-metastatic bladder cancer undergoing radical cystectomy

I (intervention): RARC with or without totally intracorporeal urinary diversion (ICUD vs. ECUD)

C (comparator): ORC

O (outcome): Primary endpoints were 90-day complications, operative time, estimated blood loss, time to bowel recovery, length of stay, transfusion rate and QoL assessment.

S (study design): Randomized Controlled Trials

### 2.2. Study Selection

We included prospective RCTs comparing RARC and ORC for bladder cancer. Two authors (E.B. and L.C.L) performed the initial screening—independently—of all published manuscripts. Any disagreement was discussed with a third co-author (R.S.F.) and resolved by consensus. Data extraction was verified for accuracy by another reviewer (F.P.) before the statistical analysis.

We collected study characteristics including author, year, country, number of patients included, enrollment period, primary and secondary outcomes evaluated, patient demographic characteristics, type of urinary diversion, neoadjuvant chemotherapy, lymph nodes template, pathological T stage and adjuvant chemotherapy.

Perioperative outcomes include estimated blood loss (EBL), blood transfusion rate, operative time (OT), length of stay (LOS), time to bowel recovery, 90-day complications—defined using the Clavien-Dindo (CD) Classification [18]—and are stratified into minor (CD I-II) and major (CD III-V) complications. Quality of life outcomes included all domains of the EORTC QLQC-30 [19] and were stratified into functional domains and symptoms domains.

### 2.3. Risk of Bias Assessment

The risk of bias (RoB) was assessed using the Cochrane Risk-of-Bias Tool for randomized trials (RoB 2.0) [20]. The RoB was evaluated only for operative outcomes, complications, and quality of life. The RoB graphic was created using the {robvis} package in R software (ver. 4.3.1), and it is provided in the Supplementary Material (Figures S1 and S2).

### 2.4. Statistical Analysis

Meta-analyses were performed when two or more studies reported the same outcomes (RARC vs. ORC) under the same definition. Means and standard deviations (SDs) or medians and interquartile ranges (IQRs) or medians and ranges were utilized for continuous variables. For primary studies reporting only the median and IQR, or the minimum-to-maximum range, the Box-Cox method for estimating the sample mean and standard deviation [21] were utilized. The number of events as a proportion of the sample size was collected for dichotomous variables.

We assessed heterogeneity using the Cochran’s Q-test [22], and the I^2^ statistic was used to describe the proportion of interstudy variation caused by heterogeneity, with an I^2^ value of 0–40% considered to represent negligible heterogeneity, 30–60% to represent moderate heterogeneity, 50–90% to represent substantial heterogeneity, and 75–100% to represent considerable heterogeneity.

For outcomes with moderate heterogeneity and higher, a random-effect model (by DerSimonian and Laird) was used to obtain pooled estimates. Otherwise, a fixed-effect model (Mantel-Haenszel) was used for dichotomous variables and the inverse-variance (I–V) model was used for continuous variables. For the assessment of QoL, we conducted a pooled analysis of the standardized mean difference comparing the two approaches before and after the intervention. This enabled us to evaluate and compare the average results across different domains.

We then utilized radar plots to illustrate these findings, categorizing them into symptom domains and functional domains, as per the questionnaire’s structure. The pairwise meta-analysis was performed using package {metafor} R software (ver. 4.3.1) [23].

Statistical significance was set at a *p*-value < 0.05.

Random-effects network meta-analysis was performed using a frequentist framework, implemented in the {netmeta} package for R [24]. The analyzed results were visualized as forest plots, net splitting forest plots, and net graphs.

## 3. Results

### 3.1. Baseline Characteristics

After a systematic review of the literature (Supplementary Materials—Table S2), eight RCTs run either in North America or Europe from 2010 to 2022 were analyzed [9,10,25,26,27,28,29,30]. Of those, two were multicenter and six were single center. Overall, 1024 patients were identified. Median age, BMI and neoadjuvant chemotherapy rates ranged from 64 to 70 years, from 26 to 29 m^2^/kg and from 10 to 45%, respectively. Baseline characteristics were comparable among all included studies, with the exception of urinary diversion (Table 1 and Table 2). Indeed, four studies compared ORC with ECUD-RARC vs. three studies compared ORC with ICUD-RARC.

### 3.2. Perioperative Outcomes: RARC vs. ORC

**Figure 1:**
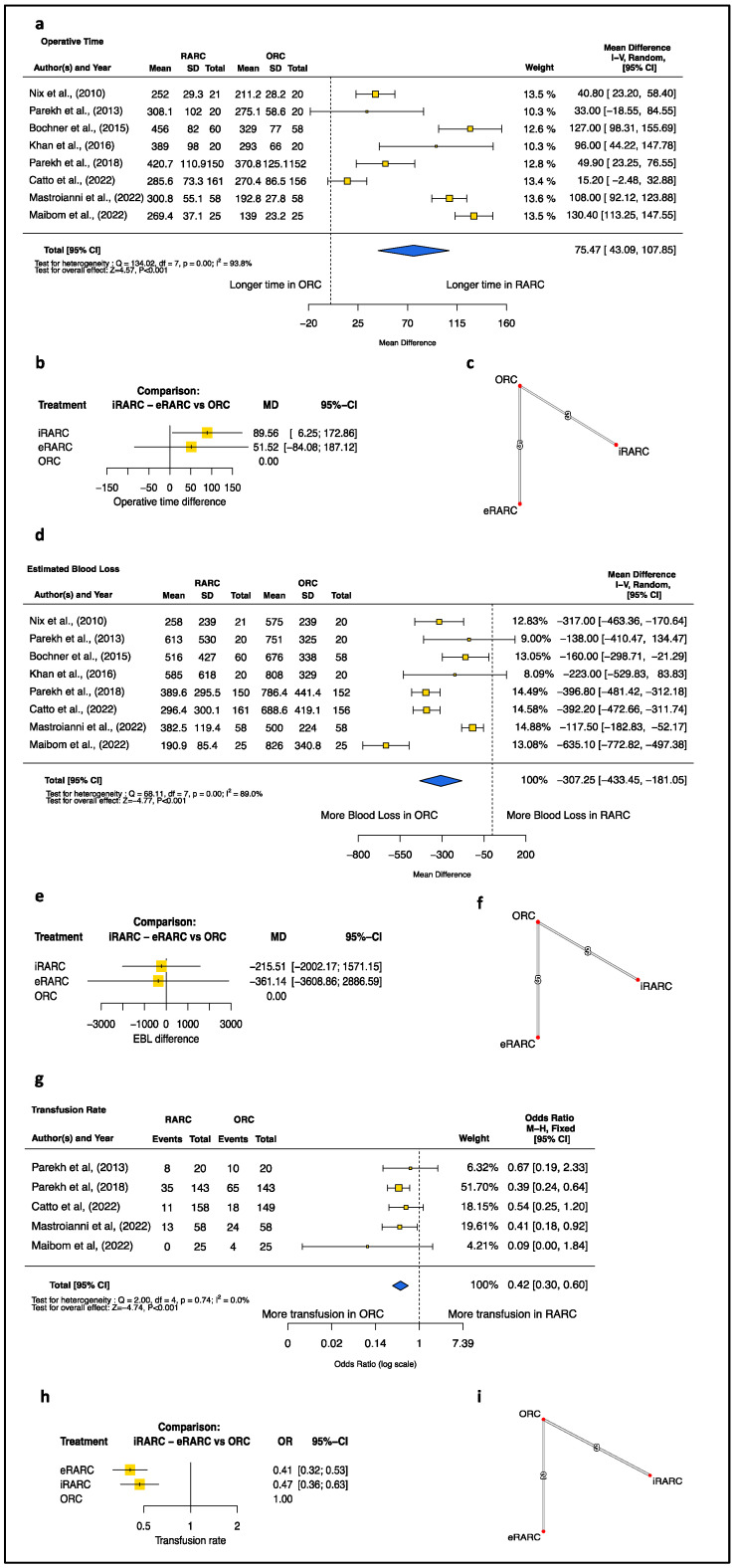
Operative Time. (**a**) Pairwise meta-analysis of the robotic versus open technique. (**b**) Network meta-analysis depicted as mixed evidence (indirect + direct evidence). (**c**) Network of included comparisons. Estimated blood loss. (**d**) Pairwise meta-analysis of the robotic versus open technique. (**e**) Network meta-analysis depicted as mixed evidence (indirect + direct evidence). (**f**) Network of included comparisons. Transfusion Rate. (**g**) Pairwise meta-analysis of the robotic versus open technique. (**h**) Network meta-analysis depicted as mixed evidence (indirect + direct evidence). (**i**) Network of included comparisons. CI = confidence interval; df = degree of freedom; SD= standard deviation [9,10,25,26,27,28,29,30].

**Figure 2:**
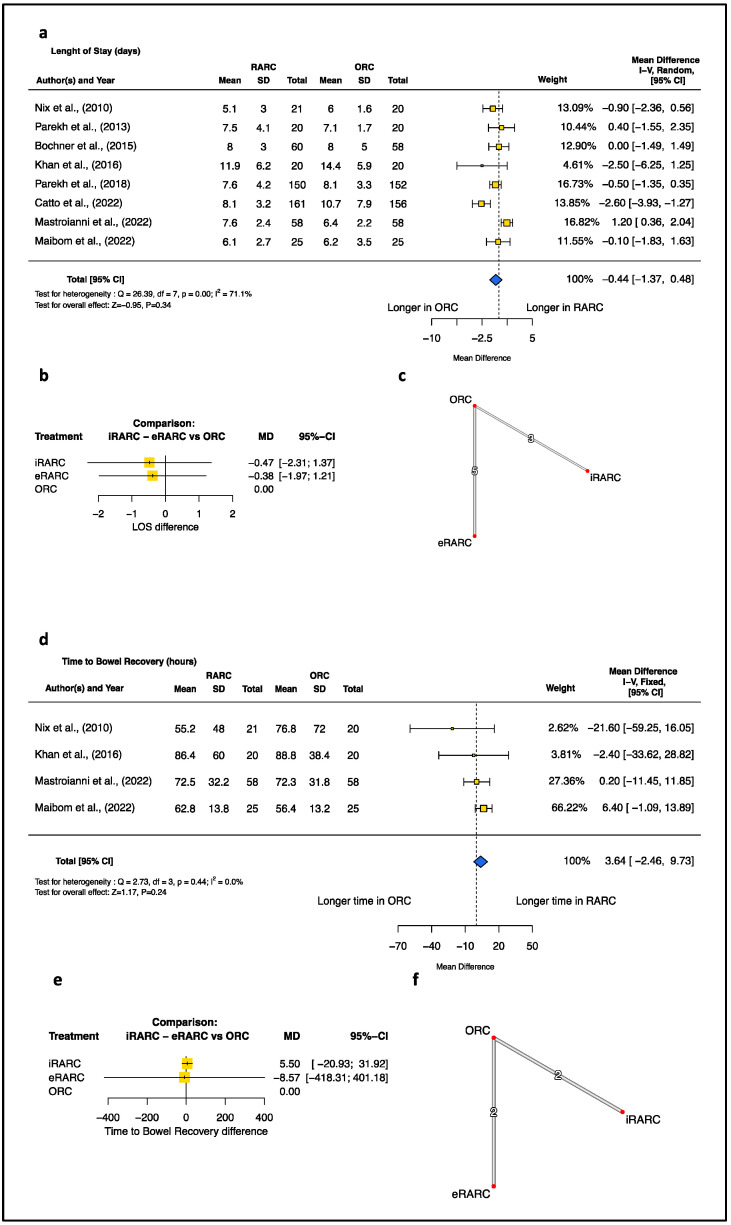
Length of Stay. (**a**) Pairwise meta-analysis of the robotic versus open technique. (**b**) Network meta-analysis depicted as mixed evidence (indirect + direct evidence). (**c**) Network of included comparisons. Time to bowel recovery. (**d**) Pairwise meta-analysis of the robotic versus open technique. (**e**) Network meta-analysis depicted as mixed evidence (indirect + direct evidence). (**f**) Network of included comparisons. CI = confidence interval; df = degree of freedom; SD = standard deviation [9,10,25,26,27,28,29,30].

**Figure 3:**
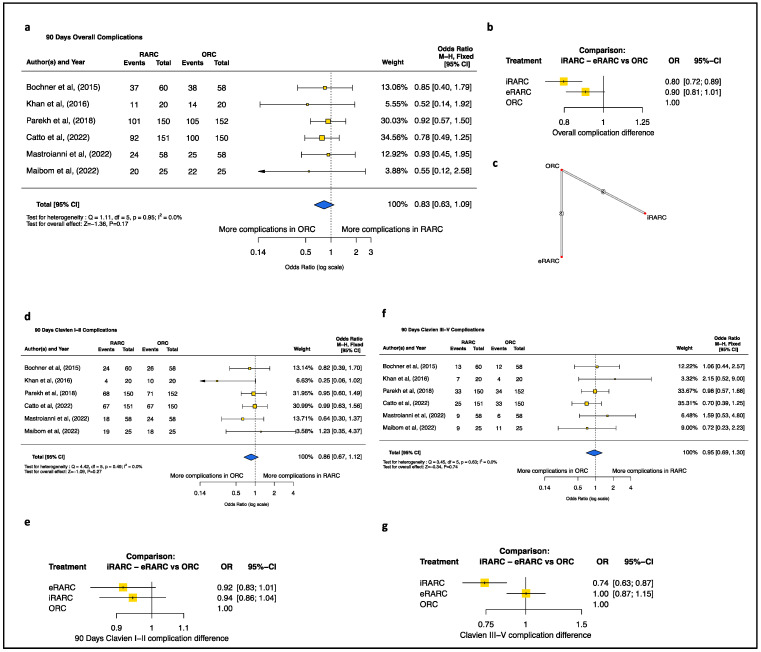
Ninety-Days Overall Complication. (**a**) pairwise meta-analysis of the robotic versus open technique. (**b**) Network meta-analysis depicted as mixed evidence (indirect + direct evidence). (**c**) Network of included comparisons. Clavien I-II 90 days Complication. (**d**) Pairwise meta-analysis of the robotic versus open technique. (**e**) Network meta-analysis depicted as mixed evidence (indirect + direct evidence). Clavien III-V 90-day Complication. (**f**) Pairwise meta-analysis of the robotic versus open technique. (**g**) Network meta-analysis depicted as mixed evidence (indirect + direct evidence). CI = confidence interval; df = degree of freedom; SD = standard deviation [9,10,27,28,29,30].

The results of the standard meta-analysis of perioperative outcomes revealed that RARC exhibited longer OT (minutes) (MD [95%CI]: 75.47 [43.09; 107.85], *p* < 0.001), lower EBL (mL) (MD [95%CI]: −307.25 [−433.45; −181.05], *p* < 0.001), and a lower transfusion rate (OR [95%CI]: 0.42, [0.30; 0.60], *p* < 0.001) than oRC (Figure 1). Conversely, no differences were detected in LOS or time to bowel recovery (Figure 2). Additionally, no difference was recorded at 90-day complications, even when considering the severity of post-operative complications according to the Clavien-Dindo classification (Figure 3).

### 3.3. Perioperative Outcomes: ECUD vs. ICUD vs. ORC

Considering direct and indirect evidence, we confirmed lower transfusion rates of both ECUD (OR [95%CI]: 0.41 [0.32; 0.53], *p* < 0.001) and ICUD (OR [95%CI]: 0.47 [0.36; 0.63], *p* < 0.001) relative to ORC (Figure 1). By contrast, only ICUD (MD [95% CI]: 89.56 [6.25; 172.86], *p* = 0.035), but not ECUD (MD [95% CI]: 51.52 [−84.08; 187.12], *p* = 0.46) exhibited a statistically significant longer OT than ORC, whereas no statistically significant differences in EBL were detected between the three approaches (Figure 1).

When considering direct and indirect evidence for endpoints—showing no differences at standard meta-analysis such as LOS, time to bowel function, and 90-day complications (Figure 2 and Figure 3)—we observed that ICUD (OR [95%CI]: 0.80 [0.72; 0.89], *p* < 0.001), but not ECUD (OR [95%CI]: 0.90 [0.81; 1.01], *p* < 0.0679), was associated with a statistically significantly lower rate of 90-day complications relative to ORC. This difference was more pronounced when considering 90-day high-grade complications for ICUD (OR [95%CI]: 0.74 [0.63; 0.87], *p* = 0.0003) and was not observed when addressing low-grade 90-day complications for ECUD (OR [95%CI]: 0.92 [0.83; 1.01], *p* = 0.08) and for ICUD (OR [95%CI]: 0.94 [0.86; 1.04], *p* = 0.27) (Figure 3). Meanwhile, no advantage emerges in terms of LOS reduction when comparing the ICUD (MD [95%CI]: −0.4745 [-2.3146; 1.3656], *p* = 0.6133) and ECUD (MD [95%CI]: −0.3830 [−1.9748; 1.2088], *p* = 0.6372) approaches with ORC.

### 3.4. Quality of Life Assessment: RARC vs. ORC

**Figure 4:**
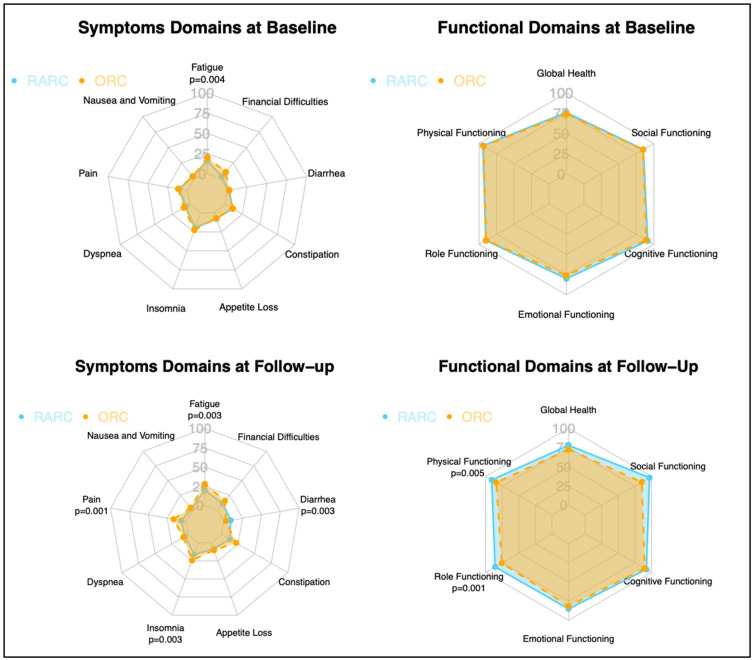
Radar plot depicting RARC and ORC according to the estimated pooled means of symptoms and functional domains of the EORTC QLQ-C30 and the comparisons of the standardized mean difference between the two approaches before and after surgery.

In our quality-of-life assessment, we included randomized studies that evaluated patients’ QoL pre- and post-intervention using the validated EORTC QoL Questionnaire Core 30 (QLQ-C30) survey. Therefore, three randomized studies were incorporated. Despite minimal differences at baseline domains (fatigue = 21.8 vs. 17.07; *p* = 0.004) (Figure 4), after surgery (3–6 months follow-up), RARC patients yielded better scores in the following symptoms domains: fatigue (28.1 vs. 20.0, *p*= 0.003), insomnia (23.8 vs. 15.4, *p* = 0.003) and pain (16.5 vs. 8.4, *p* = 0.001), as well as in the following functional domains: physical functioning (81.7 vs. 88.5, *p* = 0.005) and role functioning (75.6 vs. 84.5, *p* = 0.001). The low number of studies did not allow for direct and indirect comparisons in addition to standard metanalyses.

## 4. Discussion

Our meta-analysis encompassed eight RCTs that compared RARC with ORC. Specifically, five RCTs compared ORC vs. ECUD [9,25,26,27,28] and three RCTs compared ORC vs. ICUD [10,29,30]. This allowed for a comprehensive analysis, aiming to delineate differences in perioperative outcomes and QoL assessment between these surgical approaches.

Our analysis—aligned with previous reports [31,32]—confirms that RARC yields better outcomes regarding EBL and fewer intra- and post-operative transfusions, but longer OT compared to ORC. However, considering indirect evidence, the ICUD approach required longer OT (MD = 52, *p* = 0.46) but does not demonstrate a significant difference in OT compared to the ECUD approach. It must be emphasized that such results come from RCTs run at high-volume centers; thus, greater differences are expected when adopting this technique since OT mainly relies on the surgeon learning curve [33]. Taken together, it may be postulated that the approaching reconstructive phase intracorporeally may significantly increase OT.

A retrospective study by Bertolo et al. confirmed the ICUD approach as more time-consuming compared to ECUD (7 vs. 6 h, *p* = 0.0004) [34]. Even if these results are confirmed by randomized studies, it would be appropriate to evaluate whether the statistically significant advantage in OT could be converted into a clinically significant benefit sufficient to counterbalance the potential advantages lost from an entirely minimally invasive surgical procedure.

In this context, it is interesting to discuss how our study does not report any significant differences in terms of LOS between the two surgical approaches, both in the standard meta-analysis (MD −0.44, *p* < 0.34) and when stratifying by the urinary diversion technique in NMA. These findings are in contrast with previous reports that indicated an advantage of RARC over ORC in reducing the postoperative LOS [8,35]. Recently, Khetrapal et al., by meta-analyzing the same studies, reported a tiny difference in favor of RARC (MD 0.21 95%CI 0.03–0.39) [36]. Although the authors supported the relevance of this finding, we believe that such a difference is not clinically meaningful. Moreover, it is important to underline that some RCTs report medians and quartiles rather than mean and standard deviation. In this scenario, different published formulas can be used to obtain the mean and standard deviation from the median and quartiles. Thus, the use of a different formula may explain the discrepancy between our and their LOS results. However, we are confident in our claim that LOS differences are often overlooked since they are influenced by multiple factors, such as socio-demographic characteristics, hospital organization and enhanced recovery protocol [37]. Therefore, it can be postulated that the implementation of the ERAS protocol in most recent RCTs has leveled the tiny difference in LOS between RARC and ORC [38].

Indeed, the three most recent studies [10,29,30] explicitly report the application of the ERAS protocol, with one also providing specific data about the percentage of patients adhering to the protocol [29], while the others do not explicitly mention this information. Thus, both urologists and patients should be aware that no definitive statistically significant difference exists in terms of LOS between RARC and ORC, particularly when the ERAS protocol is implemented. Moreover, we observed no differences in time to bowel recovery between RARC and ORC and, more specifically, between ICUD, ECUD and ORC. This result is counterintuitive since no exposure to room air or hand manipulation of the bowels in ICUD was expected to seed-up its restoration [39]. However, the implementation of the ERAS protocol, prospective data collection and randomized design have potentially minimized confounders, resulting in a negligible impact of ICUD on bowel recovery.

Despite the lack of statistically significant differences in perioperative morbidity between RARC and ORC, we recorded an unexpected difference between ICUD and ORC for overall and high-grade 90-day complications. Dissecting raw data, we hypothesize that this difference is mainly driven by the low proportions of high-grade 90-day complications reported by Catto et al. (16.6%) [10] and Mastroianni et al. (15.5%) [29] in the ICUD arm relative to the pooled ORC arm (22%). Although none of these RCTs was initially powered to investigate this issue, the iROC trial by Catto et al. used a surrogate primary endpoint for complications, defined as “median number of days alive and out of the hospital within 90 days of surgery,” and reported a benefit for ICUD vs. ORC (adjusted difference, 2.2 days [95% CI, 0.50–3.85]; *p*  =  0.01). In consequence, despite cautions in interpreting these results, ICUD instead of ECUD may contribute to limiting the burden of postoperative high-grade complications in patients undergoing radical cystectomy. Notably, these results are in contrast with a recent propensity score-matched analysis comparing perioperative outcomes between ICUD and ECUD from the International Robotic Cystectomy Consortium (IRCC) [40]. Here, the authors observed that ICUD was associated with more overall complications and readmissions compared to ECUD, but not high-grade complications. The contrasting results may be easily explained by the fact that RCTs with ICUD were performed by expert surgeons, likely on top of the steep learning curve. Conversely, data from the prospectively maintained IRCC database may be affected by the heterogeneity of every-day clinical practice (including training, fellowship programs, patient selection); thus, increasing the perioperative morbidity of the more complex procedure, namely ICUD. Additionally, it is important to consider that thromboembolic events and wound-related complications could be the main post-operative complications driving these trends in favor of ICUD, as reported by Catto et al. [10]. Since most of the trials included in our study do not consider peri- and post-operative morbidity as primary outcomes and the reported complications are often categorized according to the Clavien-Dindo classification, we can only hypothesize that these two complications are the most responsible for the trends in favor of ICUD reported in our NMA.

Finally, we identified five RCTs comparing QoL between ORC and RARC. Of those, we excluded the two studies from Parekh et al. [9,26] because they used the FACT-VCI questionnaire. Conversely, we included all the studies that relied on the EORTC QLQ-C30 survey and reported aggregated data for each domain (Bochner et al., Mastroianni et al. and Maibon et al.) [10,27,29], but not the ones providing only the overall score (Catto et al.) [10]. Interestingly, our results support an improved QoL among RARC patients at early follow-up, despite no relevant difference at baseline between RARC and ORC (Figure 4). Moreover, at one-year follow-up, Mastroianni et al. observed that patients receiving ORC were more likely to experience a decline in role functioning and higher symptoms scores than their RARC counterparts [41]. Considering the overall EORTC QLQ-C30 score, Catto et al. did not observe any differences at baseline and 26 weeks, whereas RARC yielded higher scores at both 5 (*p*  <  0.001) and 12 weeks (*p* =  0.01) [10]. Taken together, the evidence suggests that RARC may provide some short-term benefits in terms of QoL by mitigating the impact of a life-changing intervention such as radical cystectomy, but these benefits may not be sustained over time. In consequence, more research is needed to determine the long-term effects of RARC on QoL.

Several limitations must be acknowledged in interpreting the results of this meta-analysis. While heterogeneity in study selection was not a primary issue, aspects such as the surgeon’s experience, applied techniques, and different institutional protocols and methodologies might have impacted the surgical outcomes, especially considering the OT and EBL. It’s worth highlighting that four of these studies had relatively limited cohorts, with fewer than 50 patients enrolled in the respective trials. The inability to blind participants and personnel rendered all included studies vulnerable to performance bias. The foundational evidence for our research exclusively comes from RCTs, and the demographic was restricted to participants from Europe or the USA, casting doubts on the extrapolation of our conclusions to a global scale. Factors like accessibility to specific equipment, variations in health care structures, and the predilections of patients could hinder the universal relevance of our findings.

## 5. Conclusions

In conclusion, RARC represents a safe and feasible option to reduce perioperative bleeding during radical cystectomy. Notably, our results suggest that performing ICUD instead of ECUD may help reduce the burden of 90-day complications relative to ORC. Nonetheless, surgeons should be aware of the extended operative time and step-learning curve of ICUD. Finally, RARC may provide some short-term benefits in terms of QoL, but more research is needed to determine its long-term effects.

## Figures and Tables

**Table 1:** General information and characteristic of the included studies.

Author	Study Design	Year	Country	N. of Patients per Group (n)		UD	Age (Years)		Male (%)		BMI	
				RARC	oRC		RARC	oRC	RARC	oRC	RARC	oRC
Nix et al. [25]	Single-center RCT	2010	USA	21	20	ECUD	67.4 (33–81) ^a^	69.2 (51–80) ^a^	66.7	85	27.5 (−) ^b^	28.5 (−) ^b^
Parekh et al. [26]	Single-center RCT	2013	USA	20	20	ECUD	69.5 (62.3–74) ^c^	64.5 (59.8- 72.3) ^c^	90	80	27.6 (24.2–29.9) ^c^	28.3 (26.1–32.3) ^c^
Bochner et al. [27]	Single-center RCT	2015	USA	60	58	ECUD	66 (60–71) ^c^	65 (58–69) ^c^	85	72	27.9 (24.7–31.0) ^c^	29.0 (26.3–33.7) ^c^
Khan et al. [28]	Single-center RCT	2016	GB	20	20	ECUD	68.6 (6.8) ^b^	68.6 (9.9) ^b^	85	90	27.5 (4.2) ^b^	27.4 (3.9) ^b^
Parekh et al. [9]	Multicenter RCT	2018	USA	150	152	ECUD	70 (43–90) ^c^	67 (37–85) ^c^	84	84	27.8 (25.0–30.8) ^c^	28.2 (24.9–31.7) ^c^
Catto et al. [10]	Multicenter RCT	2022	GB	161	156	ICUD	69.3 (8.0) ^b^	68.7 (8.4) ^b^	80	78	- (<18.5): 1% - (18.5–24.9): 29% - (25–29.9): 45% - (>30): 25%	- (<18.5): 3% - (18.5–24.9): 27% - (25–29.9): 46% - (>30): 4%
Mastroianni et al. [29]	Single-center RCT	2022	Italy	58	58	ICUD	64 (53–70) ^c^	66 (58–71) ^c^	76	69	26 (23–28) ^c^	26 (24–29) ^c^
Maibon et al. [30]	Single-center RCT	2022	Denmark	25	25	ICUD	70 (63–74) ^c^	67 (59–74) ^c^	72	80	27.3 (23.3–29.4) ^c^	26.9 (22.9–29.6) ^c^

RCT = Randomized Controlled Trial; UD = Urinary Diversion; RARC = Robot-Assisted Radical Cystectomy; oRC = open Radical Cystectomy; ECUD = Extra-Corporeal Urinary Diversion; ICUD = Intra-Corporeal Urinary Diversion; BMI = Body Mass Index. ^a^ Mean (Range); ^b^ Mean (SD); ^c^ Median IQR.

**Table 2:** Additional patients’ characteristic of the included studies.

Author	ASA		nCHT (%)		pT (%)		pN (%)	
	RARC	oRC	RARC	oRC	RARC	oRC	RARC	oRC
Nix et al. [25]	2.71 (mean)	2.70	-	-	≤pT2 = 66.6 >pT2 = 14.3	≤pT2 = 40 >pT2 = 25	pN+ = 19	pN+ = 35
Parekh et al. [26]	3 (median)	3	30	25	≤pT2 = 50 >pT2 = 50	≤pT2 = 65 >pT2 = 35	pN+ = 20	pN+ = 20
Bochner et al. [27]	2 = 28% 3 = 70% 4 = 1.7%	2 = 21% 3 = 74% 4 = 5.2%	31.7	44.8	≤pT2 = 71.6 >pT2 = 28.4	≤pT2 = 67.2 >pT2 = 32.8	pN+ = 17	pN+ = 16
Khan et al. [28]	1 = 20% 2 = 75% 3 = 5%	1 = 20% 2 = 75% 3 = 5%	10	15	≤pT2 = 70 > pT2 = 30	≤pT2 = 70 >pT2 = 30	-	-
Parekh et al. [9]	ECOG 0 = 78% ECOG 1 = 19% ECOG 2–3 = 3%	ECOG 0 = 72% ECOG 1 = 26% ECOG 2–3 = 3%	27.3	36.2	≤pT2 = 69.3 >pT2 = 30.7	≤pT2 = 68.4 >pT2 = 31.6	pN+ = 23	pN+ = 24
Catto et al. [10]	ECOG 0 = 81% ECOG 1 = 15% ECOG 2–3 = 3%	ECOG 0 = 81% ECOG 1 = 17% ECOG 2–3 = 2%	33.5	33.9	≤pT2 = 70 >pT2 = 30	≤pT2 = 75 >pT2 = 25	pN+ = 18	pN+ = 17
Mastroianni et al. [29]	1 = 3% 2 = 74% 3 = 22%	1 = 3% 2 = 86% 3 = 10%	39.6	37.9	≤pT2 = 63 >pT2 = 37	≤pT2 = 67 >pT2 = 33	pN+ = 13	pN+ = 14
Maibon et al. [30]	1 = 8% 2 = 72% 3 = 20%	1 = 12% 2 = 76% 3 = 12%	36	40	≤pT2 = 88 >pT2 = 12	≤pT2 = 84 >pT2 = 16	pN+ = 12	pN+ = 28

RARC = Robot-Assisted Radical Cystectomy; oRC = open Radical Cystectomy; nCHT = neoadjuvant chemotherapy. ASA = American Society of Anesthesiologists Physical Status Classification System; ECOG = Eastern Cooperative Oncology Group Performance Status; pN = Pathological Nodal Stage; pT: Pathological Tumor Stage.

## Data Availability

The data presented in this study are available upon request from the corresponding author. The data are not publicly available due to restrictions.

## Supplementary Materials

The following supporting information can be downloaded at: https://www.mdpi.com/article/10.3390/jcm13082421/s1, Table S1: Search Strategy; Table S2: Prisma Flow-Chart; Figure S1: Risk of bias Assessment and results—Perioperative Outcomes; Figure S2: Risk of bias Assessment and results—Quality of Life.
