# Supplementary material for: Comparative Outcomes of Open Radical Cystectomy vs. Robot-Assisted Approaches with Intracorporeal and Extracorporeal Urinary Diversion: A Meta-Analysis and Network Meta-Analysis of Perioperative and Quality of Life Outcomes
Source: J Clin Med. 2024 Apr 21;13(8):2421. doi: 10.3390/jcm13082421 (PMC11051502; doi:10.3390/jcm13082421)

Supplemetary Matherial

Supplemetary Table S1. Search Strategy

MEDLINE (PubMed) (2023-04)	1. Robot* [OR]<br>2. Robotical [OR]<br>3. Robotic [OR]<br>4. Robot Assisted [OR]<br>5. Robot-Assisted [OR]<br>6. Minimally invasive [OR]<br><br>[AND]<br><br>7. Open [OR]<br>8. Conventional [OR]<br>9. Classic [OR]<br>10. Classical [OR]<br>11. Traditional [OR]<br><br>[AND]<br><br>12. Cystectomy [OR]<br>13. Radical bladder [OR]<br>14. Cystoprostatectomy [OR]<br>15. Bladder surgery [OR]<br>16. Bladder Removal [OR]<br><br>[AND]<br><br>17. Random Allocation<br>18. random allocation<br>19. “control* clinic* trial”<br>20. “control* trial”<br>21. RCT<br>22. random*<br>23. Compar*<br>24. Trial<br><br>"(Robot* OR Robotical OR Robotic OR Robot Assisted OR Robot-Assisted OR Minimally invasive) AND (Open OR Conventional OR Classic OR Classical OR Traditional) AND (Cystectomy OR Radical bladder OR Cystoprostatectomy OR Bladder surgery OR Bladder Removal) AND (Random Allocation OR random allocation OR 'control* clinic* trial' OR 'control* trial' OR RCT OR random* OR Compar* OR Trial)"
Web Of Science (2023-04)	1. Robot* [OR]<br>2. Robotical [OR]<br>3. Robotic [OR]<br>4. Robot Assisted [OR]<br>5. Robot-Assisted [OR]<br>6. Minimally invasive [OR]

	[AND]<br><br>7. Open [OR]<br>8. Conventional [OR]<br>9. Classic [OR]<br>10. Classical [OR]<br>11. Traditional [OR]<br><br>[AND]<br><br>12. Cystectomy [OR]<br>13. Radical bladder [OR]<br>14. Cystoprostatectomy [OR]<br>15. Bladder surgery [OR]<br>16. Bladder Removal [OR]<br><br>[AND]<br><br>17. Random Allocation<br>18. random allocation<br>19. “control* clinic* trial”<br>20. “control* trial”<br>21. RCT<br>22. random*<br>23. Compar*<br>24. Trial<br><br>TS=((Robot* OR Robotical OR Robotic OR Robot Assisted OR Robot-Assisted OR Minimally invasive) AND (Open OR Conventional OR Classic OR Classical OR Traditional) AND (Cystectomy OR Radical bladder OR Cystoprostatectomy OR Bladder surgery OR Bladder Removal) AND (Random Allocation OR random allocation OR 'control* clinic* trial' OR 'control* trial' OR RCT OR random* OR Compar* OR Trial))

Scopus (2023-04)	1. Robot* [OR]<br>2. Robotical [OR]<br>3. Robotic [OR]<br>4. Robot Assisted [OR]<br>5. Robot-Assisted [OR]<br>6. Minimally invasive [OR]<br><br>[AND]<br><br>7. Open [OR]<br>8. Conventional [OR]<br>9. Classic [OR]<br>10. Classical [OR]<br>11. Traditional [OR]<br><br>[AND]

	12. Cystectomy [OR]<br>13. Radical bladder [OR]<br>14. Cystoprostatectomy [OR]<br>15. Bladder surgery [OR]<br>16. Bladder Removal [OR]<br><br>[AND]<br><br>17. Random Allocation<br>18. random allocation<br>19. “control* clinic* trial”<br>20. “control* trial”<br>21. RCT<br>22. random*<br>23. Compar*<br>24. Trial<br><br>(TITLE-ABS-KEY ("Robot*") OR TITLE-ABS-KEY ("Robotical") OR TITLE-ABS-KEY ("Robotic") OR TITLE-ABS-KEY ("Robot Assisted") OR TITLE-ABS-KEY ("Robot-Assisted") OR TITLE-ABS-KEY ("Minimally invasive")) AND (TITLE-ABS-KEY ("Open") OR TITLE-ABS-KEY ("Conventional") OR TITLE-ABS-KEY ("Classic") OR TITLE-ABS-KEY ("Classical") OR TITLE-ABS-KEY ("Traditional")) AND (TITLE-ABS-KEY ("Cystectomy") OR TITLE-ABS-KEY ("Radical bladder") OR TITLE-ABS-KEY ("Cystoprostatectomy") OR TITLE-ABS-KEY ("Bladder surgery") OR TITLE-ABS-KEY ("Bladder Removal")) AND (TITLE-ABS-KEY ("Random Allocation") OR TITLE-ABS-KEY ("random allocation") OR TITLE-ABS-KEY ("control* clinic* trial") OR TITLE-ABS-KEY ("control* trial") OR TITLE-ABS-KEY ("RCT") OR TITLE-ABS-KEY ("random*") OR TITLE-ABS-KEY ("Compar*") OR TITLE-ABS-KEY ("Trial"))

Cochrane Library (2023-04)	1. Robot* [OR]<br>2. Robotical [OR]<br>3. Robotic [OR]<br>4. Robot Assisted [OR]<br>5. Robot-Assisted [OR]<br>6. Minimally invasive [OR]<br><br>[AND]<br><br>7. Open [OR]<br>8. Conventional [OR]<br>9. Classic [OR]<br>10. Classical [OR]<br>11. Traditional [OR]<br><br>[AND]<br><br>12. Cystectomy [OR]<br>13. Radical bladder [OR]<br>14. Cystoprostatectomy [OR]

	15. Bladder surgery [OR]<br>16. Bladder Removal [OR]<br><br>[AND]<br><br>17. Random Allocation<br>18. random allocation<br>19. “control* clinic* trial”<br>20. “control* trial”<br>21. RCT<br>22. random*<br>23. Compar*<br>24. Trial<br><br>(("Robot*" OR "Robotical" OR "Robotic" OR "Robot Assisted" OR "Robot-Assisted" OR "Minimally invasive") AND ("Open" OR "Conventional" OR "Classic" OR "Classical" OR "Traditional") AND ("Cystectomy" OR "Radical bladder" OR "Cystoprostatectomy" OR "Bladder surgery" OR "Bladder Removal") AND ("Random Allocation" OR "random allocation" OR "control* clinic* trial" OR "control* trial" OR "RCT" OR "random*" OR "Compar*" OR "Trial"))

Clinical Trial gov (www.clinical trials.gov/) (2023-04)	1.Robotic | Bladder Cancer | Cystectomy | Adult, Older Adult

Supplementary Table S2 .Prisma Flow-Chart

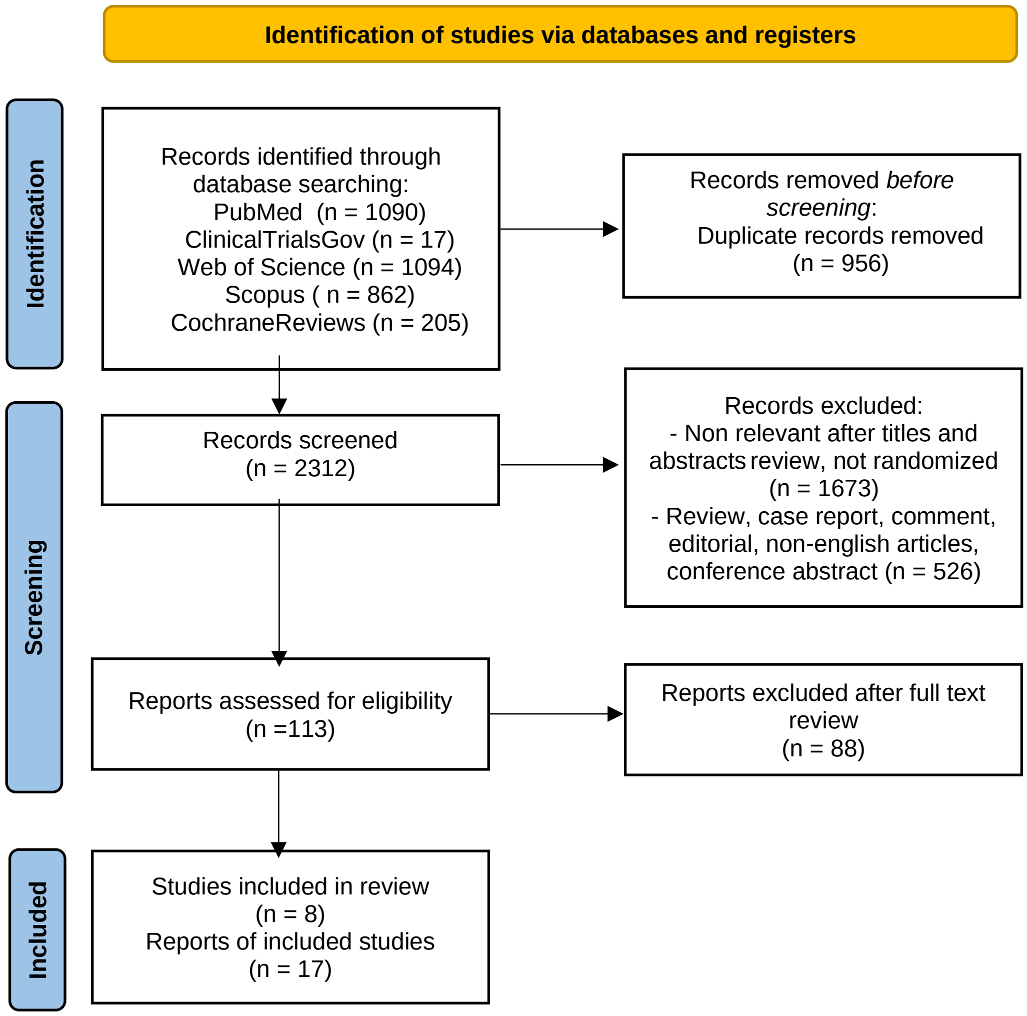

Supplementary Figure S1. Risk of bias Assessment and results – Perioperative Outcomes

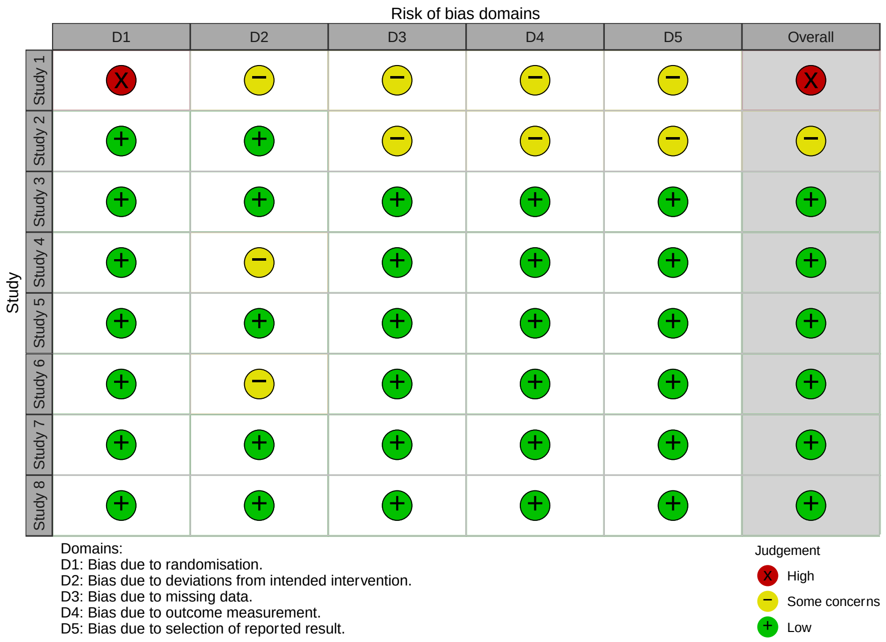

Study 1	Nix et al. (2010)
Study 2	Parekh et al. (2013)
Study 3	Bochner et al. (2015)
Study 4	Khan et al. (2016)
Study 5	Parekh et al. (2018)
Study 6	Catto et al. (2022)
Study 7	Mastroianni et al. (2022)
Study 8	Maibom et al. (2022)

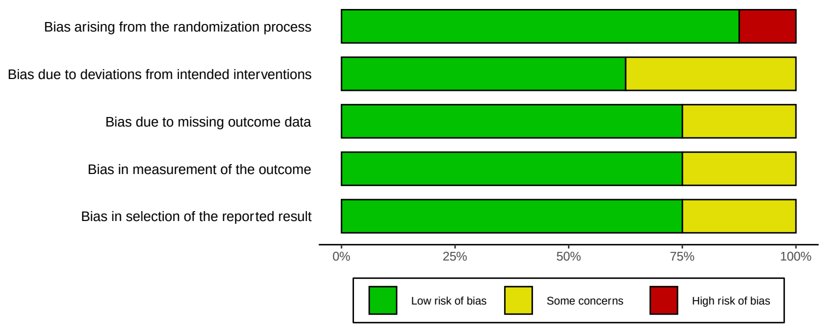

Supplementary Figure S2. Risk of bias Assessment and results – Quality of Life

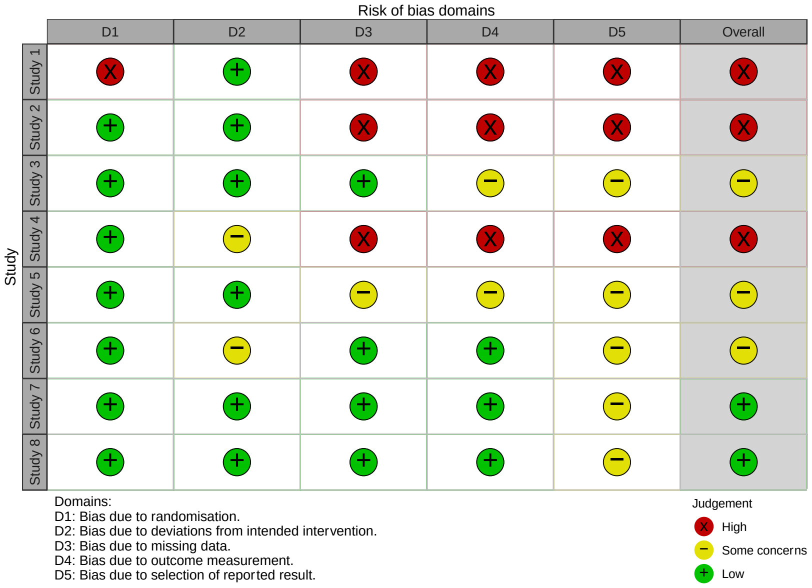

Study 1	Nix et al. (2010)
Study 2	Parekh et al. (2013)
Study 3	Bochner et al. (2015)
Study 4	Khan et al. (2016)
Study 5	Parekh et al. (2018)
Study 6	Catto et al. (2022)
Study 7	Mastroianni et al. (2022)
Study 8	Maibom et al. (2022)

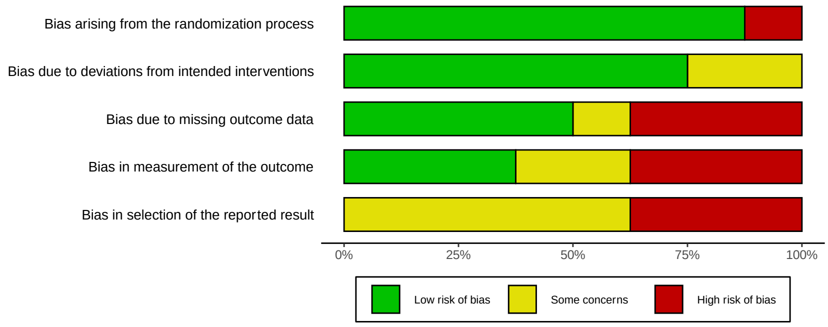

Supplement: Supplementary file 1 [file jcm-13-02421-s001.zip › jcm-2926524-supplementary.pdf]
